# Prefrontal cortical microcircuits bind perception to executive control

**DOI:** 10.1038/srep02285

**Published:** 2013-07-29

**Authors:** Ioan Opris, Lucas Santos, Greg A. Gerhardt, Dong Song, Theodore W. Berger, Robert E. Hampson, Sam A. Deadwyler

**Affiliations:** 1 Department of Physiology and Pharmacology, Wake Forest University School of Medicine, Winston-Salem, NC; 2 Department of Anatomy and Neurobiology, University of Kentucky, Lexington, KY; 3 Department of Biomedical Engineering, University of Southern California, LA, CA

## Abstract

During the perception-to-action cycle, our cerebral cortex mediates the interactions between the environment and the perceptual-executive systems of the brain. At the top of the executive hierarchy, prefrontal cortical microcircuits are assumed to bind perceptual and executive control information to guide goal-driven behavior. Here, we tested this hypothesis by comparing simultaneously recorded neuron firing in prefrontal cortical layers and the caudate-putamen of rhesus monkeys, trained in a spatial-versus-object, rule-based match-to-sample task. We found that during the perception and executive selection phases, cell firing in the localized prefrontal layers and caudate-putamen region exhibited similar location preferences on spatial-trials, but less on object- trials. Then, we facilitated the perceptual-executive circuit by stimulating the prefrontal infra-granular-layers with patterns previously derived from supra-granular-layers, and produced stimulation-induced spatial preference in percent correct performance on spatial trials, similar to neural tuning. These results show that inter-laminar prefrontal microcircuits play causal roles to the perception-to-action cycle.

A broad range of brain functions, from perceptual to executive actions encode, represent, monitor and select information that is either spatial- and/or object-specific for effective behavioral performance^1^,^2^,^3^,^4^,^5^,^6^,^7^. Such constellations of brain abilities use large scale neural circuits consisting of thalamo-cortical loops and cortical microcircuits with functional roles in the integration and selection of information^8^,^9^,^10^. The term “cognit” was coined by Fuster^8^ for such distributed functions in which the same neurons participate in several different circuits (‘bottom-up’ in parietal/temporal-to-frontal and ‘top-down’ in frontal-to-caudate/putamen or other subcortical regions)^8^.

It has been previously shown that dorsal visual stream of neural projections from the striate cortex to the posterior parietal region carries the spatial information (Figure 1A) required for sensorimotor transformations in visually guided actions, while the ventral stream projections from the striate cortex to the inferior temporal cortex is primarily responsible for perceptual identification of objects^11^,^12^. Thus, a visual object's qualities and its spatial location depend on the processing of different types of visual information in the inferior temporal and posterior parietal cortex, respectively. However, object and spatial information carried in these two separate pathways has been shown to be integrated into a unified ‘visual percept’ in prefrontal cortex which receives connections from both circuits^11^,^12^,^13^.

Several lines of evidence indicate that the basal ganglia participates in multiple parallel segregated circuits or ‘thalamo-cortical loops’ that make connections with motor, sensory and cognitive areas of the cerebral cortex^9^,^14^,^15^. Prefrontal cortical areas seem to be the target of extensive, topographically organized outputs from the basal ganglia^14^. Such thalamo-cortical projections from basal ganglia to the superficial and deep prefrontal cortical layers can directly activate specific inputs to the re-entrant loop^16^,^17^. Thus, the outputs from the inter-laminar microcircuits of prefrontal cortex are in ideal position to support the decision to act via the synchronous excitation of the constellation of circuits in the executive hierarchy^1^,^8^.

Executive control is a fundamental function of the brain that mediates the integration of perception and action during behaviorally relevant environmental events. It has been proposed that executive control involves a broad network of brain areas, including frontal and parietal/temporal cortex, as well as striatum and other subcortical structures^8^. These structures have been consistently associated with roles in sensorimotor integration and selection of task specific behavioral responses, commonly considered to be the regions necessary for ‘executive decisions’^18^,^19^. However, what is not known is how such areas are synchronously activated via the inter-laminar microcircuits that operate to segregate information in a manner consistent with control of movements necessary during the perception-to-action cycle that defines executive decision making in behavioral tasks.

## Results

**Figure 1:**
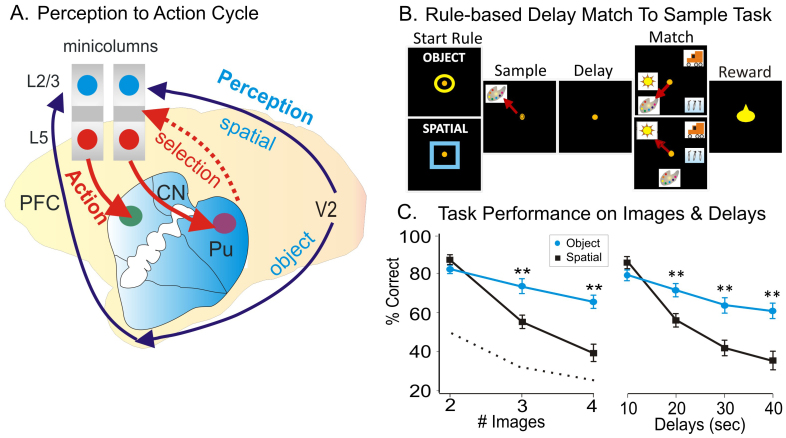
The perception-to-action cycle with the behavioral paradigm. (A). The illustration of the perception-to-action cycle. The diagram depicts the flow of spatial and object signals during perceptual and executive selection of target stimuli in a rhesus macaque brain. In visual cortical area V2 visual information splits into dorsal (spatial signals) and ventral (object signals) pathways that send signals to the top of executive hierarchy in prefrontal cortex, and then top-down through the cortico-striatal-thalamo-cortical loops. Blue arrows depict the perceptual flow of information while red arrows indicate the action (executive) signal flow from prefrontal cortical layer 5 to dorsal striatum, with the red dotted arrow indicating the thalamo-cortical projection in the cortico-striatal-thalamo-cortical loop. The two adjacent cortical minicolumns with red and blue filled circles indicate inter-laminar simultaneous recordings, while caudate-putamen recording are shown in green and pink circles. PFC-prefrontal cortex layers L2/3 and L5, and V2-secondary visual cortex region. (B). Behavioral paradigm showing the sequence of events in the rule-based DMS task. Each trial begins with ‘trial start images’ (‘ring’ or ‘box’) to initiate an ‘object’ or ‘spatial’ trial, respectively. Then, presentation of the ‘Sample Target’ image is accompanied by a ‘Sample Response’, followed by a variable ‘Delay’ period of 1–40 sec, with blank screen; followed by presentation of the ‘Match’ screen with Sample image accompanied by 1–6 Non-match (distracter) images, requiring movement of the cursor into the correct Match target determined by ‘trial start’ screen (Spatial trial = same location on the screen, or Object trial = same image-irrespective of position, responded to in the Sample phase) after presentation to receive a juice reward, via an accessible sipper tube. Placement of the cursor into a Non-match target (>0.5 s) caused the screen to blank without reward delivery. Inter-trial interval (ITI) = 10.0 s. (C). Behavioral performance in the DMS task. Behavioral performance (% correct trials) is shown separately for spatial trials (blue) vs. object trials (red) for trials ranging from 2–4 images (F(1,239) = 12.54; p < 0.001) and 1–40 sec delays (F(1,239) = 12.32; p < 0.001). Asterisks: **p < 0.001, ANOVA.

Prior evidence suggests that a critical role in this mechanism is played by inter-laminar microcircuits^20^,^21^,^22^,^23^,^24^ consisting of prefrontal cortical minicolumns^17^,^25^,^26^,^27^,^28^ which receive converging inputs in the supra-granular layers and send top-down outputs via columnar connections with sub-granular layers to dorsal striatum (caudate nucleus and putamen) to provide parallel cortico-striatal-thalamo-cortical loops^6^,^9^,^14^,^15^ for different types of sensory inputs. However, the manner in which these local groups of neurons embedded in cortico-striatal loops dynamically encode and transform population codes across cortical layers to select spatial or object information for the executive control of behavior, remains a mystery. The evidence presented here shows that these brain structures are part of functional loops (Figure 1A) in which inter-laminar microcircuits or ‘minicolumns’ in dorsolateral prefrontal cortex ‘bind’ perception and executive selection of spatial targets to guide goal-specific behavior. As stated in the past, it is only natural that the ‘critical link’ for this process, is in the operation of prefrontal minicolumns^8^. Figure 1A illustrates this perception-to-action cycle which consists of bottom-up visual pathways (dorsal visual stream for spatial and ventral stream for object information) with the associated inter-laminar prefrontal microcircuits on top of hierarchy, and top-down cortico-striatal-pallidal-thalamo-cortical loops.

One reason that supports the view that prefrontal cortical inter-laminar microcircuits at the top of executive hierarchy play an active role in linking the perceptual and executive control signals is the fact that the disruption of cortical minicolumns is present in post mortem cortical tissue from humans that suffered from autism^29^, dementia^30^ or other psychiatric disorders.

Our approach to examine neural responses relevant to the perception-to-action information cycle^1^,^8^ involves determining the integrative role of prefrontal layer 2/3^10^,^31^, the role in target selection^18^ of prefrontal layer 5 and the involvement of caudate in goal directed behavior. An intriguing aspect of this approach is the involvement of the prefrontal inter-laminar microcircuit that links the perceptual and executive/action related circuits as we have previously demonstrated^19^ in nonhuman primates. To examine how perception and executive selection of visual information operate in prefrontal cortico-striatal loops (Fig. 1A) we recorded simultaneously cell firing in the prefrontal cortical cell layers 2/3 and 5, with cell activity in the caudate/putamen in rhesus macaques. A multi-electrode array with recording locations that conformed to the microanatomy of the cortex when inserted into the supra-granular and infra-granular layers in PFC was used. This allowed simultaneous recordings from mini-columnar pyramidal cells in PFC cortical layers 2/3 & 5^18^,^19^. This was combined with a tetrode array^32^ positioned to record simultaneously cells in the caudate-putamen while animals performed a DMS short-term memory task (Fig. 1S Supplemental Information).

Four nonhuman primates (rhesus monkeys) were trained to perform a delayed match to sample (DMS) task with the instruction to select either 1) the remembered image on the screen (object trial) or 2) the spatial location of the image on the screen (spatial trial), each presented in the Sample phase of the task (Fig. 1C). In both versions of the task (Object or Spatial), subjects made hand tracking movements to the appropriate visual targets for rewards in the Match phase of the task (Fig. 1C). The DMS task incorporated key features like the number of distracter images (2–4) which could appear in any of eight locations on the screen in the Match phase after variable durations of the intervening delay period (1 to 40 sec). These factors were reflected in the animal's behavioral performance levels during encoding and selection of spatial or object stimuli as shown in Figure 1D.

**Figure 2:**
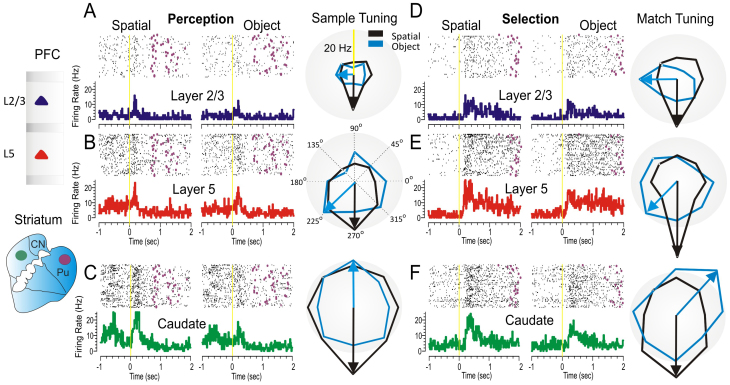
Neural firing in prefrontal cortical layers and striatum on spatial vs. object trials. (A–F). Example of simultaneous individual activity (individual trial rasters and peri-event histograms) of single neurons recorded in prefrontal cortical layers L2/3 (A, D: blue) and L5 (B, E: red) with the conformal MEA and caudate n. (C, E: green) during Sample (A,B,C) and Match (D,E,F) target presentation on Spatial (left panels) and Object (center panels) trials during a single session (n = 120 trials). The purple marks in the rasters represent the time when the target was reached. Directional tuning plots (A, B, C for perception and C, D, E for executive selection, right panels) depict firing preference, measured by the radial eccentricity (in spike/sec or Hz) in the polygonal contour for the eight different target locations on the screen where images appear. The overlay tuning plots compare firing preferences on Spatial (black arrow) vs. Object (pink arrow) trials for the same cells. The same tuning vectors also show the magnitude of firing for preferred locations during the encoding (left panel) and selection (right panel) phases of the task on Spatial and Object trials. Spatial trials tuning vectors (black) show the same preferred directionality (i.e. 270°) during the encoding and selection phases in both PFC layers and in caudate nucleus, suggesting parallel processing streams/loops through cortical minicolumns and striatum and likely through the entire thalamo-cortical loop. But when processing object information directional preference changes in the three cells tuning plots, suggesting that object information processing does not follow in the same “foot prints” as processing by the same cells on Spatial trials. The radius of polar plots is represented in Hz and tuning amplitude is measured in Hz, as well. Asterisks: **p < 0.001, ANOVA.

Neurons were recorded simultaneously in PFC (n = 58 cells in layer L2/3 and n = 49 cells in layer L5) and in the striatum (n = 52 cells, caudate and putamen) while the animals performed the DMS task. Only prefrontal cortical pyramidal cells with excitatory (no inhibitory) firing correlates to sample and match DMS task events, and that demonstrated significant spatial tuning, were included in analyses. Consistent with previous reports^18^,^19^ firing of cells in prefrontal layers and minicolumns reflected differential encoding of spatial and object trials in the DMS task. Figure 2A–F shows raster and peri-event histograms of cells recorded in prefrontal cortical layers 2/3 (A&D) and 5 (B&E), together with cells recorded simultaneously in caudate/putamen (C&F caudate). For each of these cells (Figure 2 and Figure 2S Supplementary Information) firing patterns were compared on object and spatial trials recorded during encoding of sample target's location of sample presentation on the screen (Perception), and during: a) the selection of the matching image (object trials), or b) selection of the spatial location of the sample image presentation on the screen (spatial trials), in the Match phase of the task (Selection). As indicated, both types of trials produced differential firing associated with Match phase selection behavior.

The polar plots in Figure 2 show that neurons in layer 2/3 and 5 fired similarly with caudate neurons and were synchronized and spatially tuned to the same screen locations (black arrows). However, when the same neurons fired on object trials (blue arrows) either a decrease or a direction change in tuning (firing to preferred location) occurred between the same 3 areas. When compared during match phase presentation (Match Tuning) neural tuning directions for the 3 regions were again similar on spatial trials (black arrows), but not on object trials (blue arrows), as shown previously^19^. This feature is extremely important because it dissociates spatial preference^33^ under these two trial conditions and indicates that increased firing in these particular microcircuit connections was sensitive to particular spatial locations where task-dependent responses are performed.

**Figure 3:**
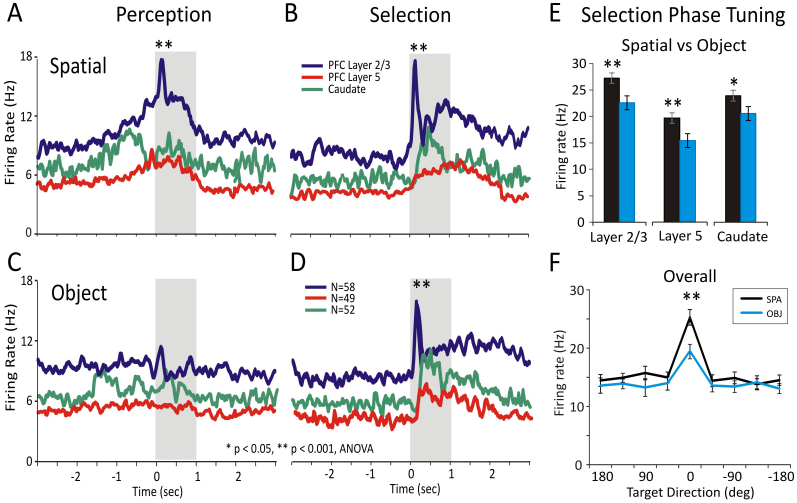
Mean firing responses and population tuning of prefrontal cortical and striatal cells during Spatial and Object trials. (A&B) Spatial trials. Comparison of mean firing rates of neurons during encoding (A) and selection (B) across prefrontal cortical layers (L23 and L5 and Striatum (Caudate nucleus) during “Spatial” trials. Prefrontal cortical L2/3 cells (n = 58) showed elevated firing during encoding and selection on spatial trials. Striatal (Caudate nucleus) cells (n = 52) showed a higher firing rates at the trial start when the spatial rule entered in effect. PFC layer 5 cells (n = 49) displayed moderate involvement in perception and selection. (C&D) Object trials. Comparison of mean firing rates of the same cells during encoding (C) and selection (D) is shown during Object trials. Cells in both prefrontal layers and striatum had much lower firing rates during Object (image) encoding and higher rates during the match, target selection, phase. The F values for (PFC layer 2/3, PFC layer L5, caudate) in (A) Sample-Spatial (F(1,1159) = 21.63, p < 0.001; F(1,979) = 6.73, p < 0.01; F(1,1039) = 7.32, p < 0.01), (B) Match-Spatial (F(1,1159) = 22.47; p < 0.001; F(1,979) = 15.56; p < 0.001; F(1,1039) = 9.13; p < 0.01), (C) Sample-Object (F(1,1159) = 1.46; p > 0.5; F(1,979) = 1.27; p > 0.5; F(1,1039) = 1.23; p > 0.5) and (D) Match-Object (F(1,1159) = 18.67; p < 0.001; F(1,979) = 16.51; p < 0.001; F(1,1039) = 14.31; p < 0.001). (E&F) Selection Phase. Comparison of neural tuning in prefrontal cortical layers and striatum during target selection on Spatial and Object trials. In (F) the arrangement of spatial locations/directions has been rotated so that the highest firing rates for all trials within the session correspond to 0° location/direction for every neuron. Error bars represent SEMs. Asterisks: **p < 0.001 ANOVA.

Figure 3A compares the average firing response during sample presentation (spatial perception) in PFC layers 2/3 and 5 with simultaneous cell firing in the striatum. In Figure 3B average firing responses of the same cells are compared during target selection in the match phase. Significant increases in firing rates of cells in PFC layers and striatum were obtained during spatial trials in both the perception (layer 2/3: F(1,1159) = 21.63, p < 0.001, n = 58; layer 5: F(1,979) = 6.73, p < 0.01, n = 49 cells; caudate: F(1,1039) = 7.32, p < 0.01, n = 52 cells; ANOVA) and selection (layer 2/3: F(1,1159) = 22.47, p < 0.001, n = 58; layer 5: F(1,979) = 15.56, p < 0.001, n = 49 cells; caudate: F(1,1039) = 9.13, p < 0.01, n = 52 cells; ANOVA) phases of the task (Fig. 3A&B), however, firing in these same areas was less during the perception phase on object trials (Fig. 3 A&C). Figure 3D shows significant increases (layer 2/3: F(1,1159) = 18.67, layer 5: F(1,979) = 16.51, caudate: F(1,1039) = 14.31, p < 0.001, ANOVA) in the firing of neurons in both PFC layers and in striatum for object trials during the target selection phase. Figure 3E&F shows a direct comparison of overall firing in the Perception and Selection phases across all 3 areas clearly indicating higher mean firing rates for spatial vs. object trials (F(1,1271) = 10.96; p < 0.001, n = 159, ANOVA; p < 0.001; Rayleigh test).

**Figure 4:**
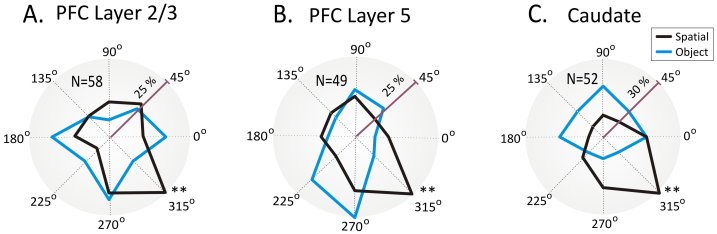
Distribution of preferred prefrontal-striatal cell firing at each target selection location. (A–C). Polar plots showing the distribution of preferred firing directions for “Spatial” and “Object” trials in PFC layer 2/3 (A), layer 5 (B) and caudate nucleus (C) recoded simultaneously during the executive selection (match) phase of the DMS task. The average % of cell firing for each cell type tuning vector direction (in Figure 2) is represented by the corresponding target location in a circular histogram. The polar plot measures the percentage of cells with highest firing rates at those locations (tuning vectors) and the asterisks indicate the highest percentage of cells from the total population with firing rates at that particular location/direction. Asterisks: **p < 0.001, Rayleigh test.

Figure 4A–C shows the polar distribution of the proportion of tuned firing across the population of cells (PFC layer 2/3, 5 and caudate) shown in Figure 3E&F, for the tuning vectors during spatial vs. object selection. Each cell's tuning vector (see Fig 2) is mapped to the corresponding target location/direction in a polar plot histogram. The overall distribution of spatial tuning shows a general preference (layer 2/3: p < 0.001, n = 58; layer 5: p < 0.001, n = 49; caudate: p < 0.001, n = 52, Rayleigh test) for the contralateral targets with only a few cells showing ipsilateral preference, as expected^34^. In addition, this distribution on signaled spatial trials was clearly distinct from that when object selection was the rule, which is consistent with the perceptual dissociation of the task^33^.

**Figure 5:**
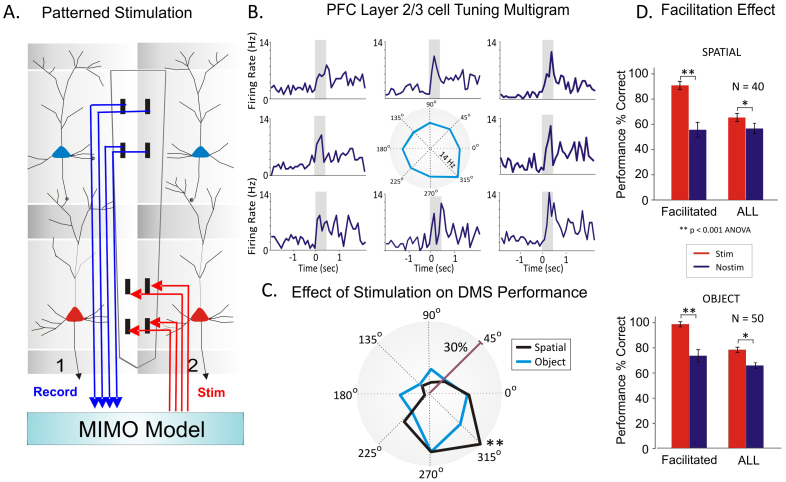
Relations of preferred target selection location to stimulation induced enhancement of cognitive performance. (A). Application of previously employed multi-input multi-output (MIMO) nonlinear model combined with the conformal MEA probe to extract the configuration of electrical (bipolar) stimulation pulses (50 uA and 1 ms) delivered to columnar recording locations in PFC layer 5^35^. (B). Peri-event multigram of a PFC layer 2/3 cell with tuning plot showing preference for the 315° target location. (C). Distribution of MIMO-stimulation facilitated correct performance locations for spatial vs. object trials across multiple sessions. Tuning vectors of percentage of correct responses on Spatial and Object trials show improved performance by the MIMO stimulation was delivered on Spatial trials in which the target was in the same position as shown in Fig. 4, for the preferred firing location (315°) of PFC and caudate neurons on nonstimulation trials. (D). Comparison of the facilitation effect of MIMO stimulation (Stim) with control (no-stim) conditions on Spatial (n = 40 sessions) and Object (n = 50 sessions) locations with the highest performance levels on Stim trials with locations of the highest performance levels on no-stim trials (Facilitated: F(1,319) = 15.34, p < 0.001 on spatial and F(1,399) = 12.68, p < 0.001 on object). These selective changes in performance produced by MIMO stimulation are shown compared with overall changes across all types of trials (ALL: F(1,319) = 6.82, p < 0.01 on spatial and F(1,399) = 9.51, p < 0.01 on object trials) in the same sessions. The differences in tuning reflected as highest % correct performance indicate that MINO stimulation also enhanced the directional preference (spatial tuning bias around 315°) of the PFC layer 2/3 recorded minicolumn (Figure 4). Error bars represent SEMs. Asterisks: **p < 0.001, ANOVA.

To further test whether inter-laminar firing links spatial perception to executive selection we applied a novel type of closed loop patterned stimulation previously shown to facilitate performance of the same task^19^,^35^. This is shown in Fig. 5 as a functional diagram in which neural firing in PFC layer 2/3 was recorded with a multielectrode array^18^,^19^ and fed into a nonlinear multi-input–multi-output (MIMO) math model (Fig. 5A & Fig. 3S Supplemental Information), which processed and simultaneously delivered a pattern of electrical pulses from a multi-channel stimulator that mimicked the correlated firing of PFC layer 5 cells on successful trials^35^. MIMO stimulation methods and associated control procedures proving columnar activation have been previously published in detail^18^,^35^. These controls included delivery of stimulation pulse patterns that were different than what the MIMO model derived for correct trials. In this case the intensity and the number of pulses, plus the area (L5) that was stimulated were identical, however the only factor that was different was the pattern that did not match the effective MIMO derived output shown in Supplementary Figure 3S.

Figure 5B shows a peri-event multigram the spatial preference firing of a PFC layer 2/3 cell during the selection phase in which the cell fired highes**t** for spatial match targets located at 315°. Figure 5C shows behavioral tuning across stimulation sessions under spatial and object rules. Spatial trials showed improved accuracy when MIMO stimulation was delivered, but performance was enhanced more on trials in which the target was in the preferred firing location (315°) on the screen (p < 0.001; Rayleigh test). This puts neural (Fig. 4) with behavioral ‘tuning’ in good agreement as it is necessary for causal relation to the perception-to-action cycle.

The effectiveness of MIMO stimulation delivered to this particular region of PFC is shown in Figure 5D where the preference effect on stimulated (Stim) vs. nonstim trials is compared for all Spatial (n = 40 sessions) and Object type trials (n = 50 sessions) within the same session. The difference in mean % correct performance for all stim vs. nonstim trials (ALL) is shown in comparison to stim vs. nonstim trials in which performance at locations was significantly above that at all other locations (Facilitated). The marked difference (F(1,319) = 13.59, p < 0.001; ANOVA) in the degree of increase in % correct trials produced by MIMO stimulation at preferred vs. non-preferred (ALL) locations indicates that in addition to facilitating performance at all response locations, the stimulation enhanced the innate directional preference (spatial tuning) which corresponded to the anatomic location of the PFC layer 2/3 minicolumn. This demonstrated that the MIMO stimulation delivered during the match/selection phase of the task was likely to have facilitated discharge of Layer 5 neurons in the same recorded minicolumns and that is what improved spatial target selection in this phase of the task.

**Figure 6:**
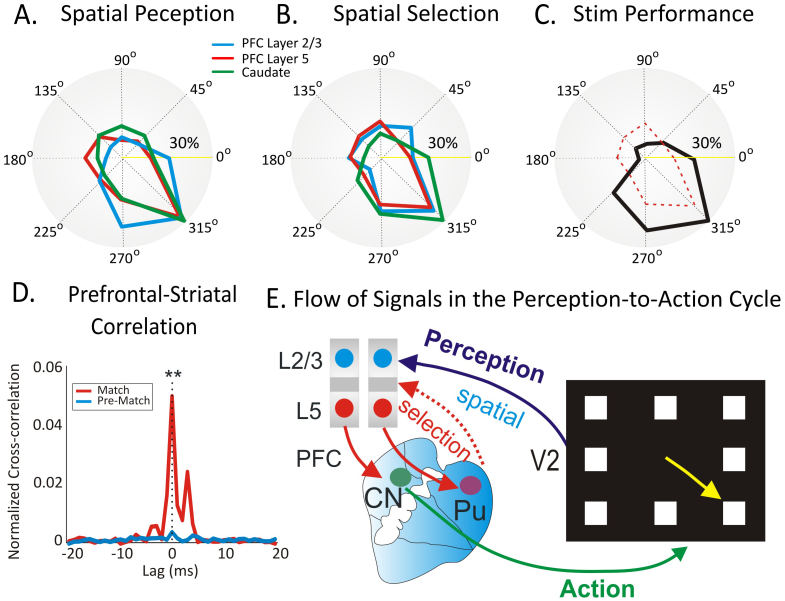
Overlap of preferred firing and stimulation induced performance tuning during the perception-to-action cycle. (A), (B). Polar plots showing the distribution of preferred firing direction for “Spatial” trials in PFC layer 2/3, layer 5 and caudate during the perception phase (A), and the executive selection phase (B) as shown in Figure 4A–C. (C). Distribution of facilitated correct performance for spatial selection during MIMO stimulation sessions (Figure 5D). The red dotted contour of tuned activity of the neurons from PFC layer 5 is overlaid to indicate similar preferred locations for columnar tuning and MIMO-stimulation facilitated performance. (D). Prefrontal-striatal correlation. Normalized cross-correlations (overlay) between n = 54 pairs of cells in PFC layer 5 and Caudate depict synchronized firing during Match target presentation (0, 2 s; red) compared to the pre-Match epoch (−2 s, 0; blue). There was a marked difference between CCHs in Match vs Pre-Match conditions; F(1,107) = 21.82, p < 0.001; ANOVA. (E). Functional diagram showing a representation of the flow of information in the PFC-caudate tuned spatial relationship across brain regions and behavior in the perception-to-action cycle. Same symbols apply as in Fig. 1 A. Asterisks: **p < 0.001, ANOVA.

The unique feature of these experiments is that they allow us to tap into the perception-to-action cycle^1^. As a final validation of microcircuit tuning in PFC and caudate we compared polar firing across the same three nodes in the perception and selection phases on spatial trials in which MIMO stimulation induced increases in performance. Figure 6 A–C shows nearly complete overlap (between 81% and 91%) in spatially tuned firing indicating that the majority of neural tuning vectors for the preferred microcircuit target location (315°) facilitated task performance when subjected to MIMO stimulation during spatial trials. The anatomic link between prefrontal cortex and striatum is demonstrated physiologically normalized cross-correlations pairs of cells in PFC layer 5 and Caudate displaying synchronized firing during Match target presentation epoch (0, 2 s; red) compared to the pre-Match epoch (−2 s, 0; blue). Therefore, such synchronized firing of PFC and Caudate neurons during the match phase (dealing with target selection and executive control; Fig. 6E) is telling us that these key nodes in the prefrontal cortical striatal loop show the modulation of executive control signals in the cortical-striatal executive loop^9^.

## Discussion

These novel findings demonstrate a robust involvement of cortical layers and striatum in the perception-to-action cycle^1^,^8^. This is supported by implementation of the MIMO model which extracts the percept from prefrontal layer 2/3 and imparts the appropriate signal to columnar related layer 5 cells, thereby strengthening activation via the executive loop through the caudate nucleus (shown in Fig. 6D) to manifest selection of a particular target location. Given these findings, the functional specificity of the perceptual circuit is likely determined via “tuned” inter-laminar microcircuits connected to executive prefrontal cortico-striatal, thalamo-cortical loops, that are translated into action via “cognits” that coordinate information in large scale networks^8^,^9^,^36^.

The enhancement in cognitive performance by the MIMO stimulation may be explained by induced changes in the balance between excitation and inhibition in cortical-striatal loop^35^,^37^ and by the temporal specificity of the PFC layered L2/3–L5 firing pattern^35^, since stimulation in a “scrambled” (random) pattern with the same pulses impaired performance in prior studies^18^,^35^. The microstimulation current activates the neighboring minicolumns around the microelectrode pad/tip causing the preference of this group of minicolumns to win the competition for the behavioral output^37^. Consequently, the memory attractor for the encoded target may recruit more relevant inputs when stimulated compared to non-stimulated control trials^38^. This view is supported by the fact that in the case of anti-phase stimulation^35^, the % correct performance decreases below the normal (non-stimulation) level possibly because some of the attractors may become repellers^39^ under that condition. We do not exclude the potential limitations of the electrical microstimulation, with respect to specificity of the effects, compared to optogenetics (for example), but the application of such methods to primates has not been accomplished yet.

Furthermore, perception and action seem consistent with a laminar segregation in gamma (40–60 Hz) and alpha (6–16 Hz) frequency coherence along the ventral stream, in which gamma coherence is confined to supra-granular layers and the alpha range to infra-granular layers^40^. Similarly, in prefrontal cortex, rule-specific synchrony at "beta" (19–40 Hz) frequencies, suggests that synchrony of beta-frequency selects the relevant rule circuit, while alpha-frequency synchrony deselects a stronger, but currently irrelevant, ensemble in complex overlapping circuits^41^. Therefore, these results clearly indicate the need for inter-laminar microcircuits to bind perception and action.

In summary, these experiments provide support for the cortical-ganglia loop model of executive control in key nodes of the loop including PFC layer 2/3, layer 5 and caudate nucleus, as well as a causal relationship involving the inter-laminar microcircuits of prefrontal cortex in tuned behavior. The results show that neuronal firing in supra-granular layers of prefrontal cortex increased during the perception phase of spatial vs. object trials while during the executive selection phase of the task both prefrontal layers and striatal cells show increases in firing rates on both types of trial (Fig. 2 & Fig. 2S). Model generated MIMO stimulation of layer 5 cells with a pattern of pulses derived from cell firing in layer 2/3 increased correct performance during selection of a spatial target at a particular spatial location during the session in which selection of targets at other locations was not improved as much. These findings suggest that prefrontal inter-laminar microcircuits play a causal role in linking perception to the executive selection of spatial targets (Fig. 6) that occupy the domain to which such microcircuitry has been tuned via past experience. In fact we were able to demonstrate activation of an innate PFC minicolumnar bias via MIMO model-controlled stimulation which resulted in improved performance on trials in which that specific type of information was required but only within a particular context. This discovery provides an important basis for building cognitive prosthetics^42^ in order to reverse cognitive deficits in a broad spectrum of diseases like schizophrenia^43^, dementia^30^, autism^29^,^44^, ADHD^45^, addiction^46^, aging^47^ and executive dysfunction^48^,^49^ in which inter-laminar processing is likely disrupted due to cortical tissue damage or malfunction^18^,^44^,^48^,^49^.

## Methods

Four male rhesus monkeys (Macaca mulatta) were utilized as subjects in all DMS sessions. Single neuron activity was recorded simultaneously from PFC and striatum (Caudate) using our tetrode microdrive^32^ and a costumed-designed multi-electrode array (MEA) specifically designed for this purpose (Center for Microelectrode Technology – CenMet, Lexington, KY)^50^. Each nonhuman primate was trained to perform a complex DMS task for juice rewards^18^,^19^. Assessment of neuron activity within different PFC cortical layers and striatum was performed using recording activity (Figs. 1S & 2S Supplementary Information) and MIMO stimulation^35^ (Fig. 3S Supplementary Information) related to Match phase image presentation up until completion of the motor target selection response, as shown in Figs. 2, 3, 5 and 2S (Supplementary Information). All surgical and animal care procedures were performed in accordance with National Institutes of Health guidelines and were approved by the Wake Forest University Animal Care and Use Committee. Full Methods and associated citations are available in the Supplementary Material file associated with this manuscript.

## Author Contributions

I.O., R.E.H. and S.A.D. designed experiments, I.O. analyzed data, I.O. and S.A.D. wrote the paper; L.M.S., I.O. and R.E.H. conducted and supervised experiments, G.A.G. provided the multi-electrode array for inter-laminar recording and microstimulation experiments, D.S. and T.W.B. provided the MIMO model and technical support for microstimulation experiments.

## Supplementary Material

Supplementary Information Supplementary Info

## References

[b1] Quintana J. & Fuster J. M. From perception to action: Temporal integrative functions of prefrontal and parietal neurons. Cerebral Cortex 19, 213–221 (1999). 10355901 10.1093/cercor/9.3.213

[b2] Goldman-Rakic P. S. The prefrontal landscape: implications of functional architecture for understanding human mentation and the central executive. Philos. Trans. R. Soc. Lond B Biol. Sci. 351, 1445–1453 (1996). 8941956 10.1098/rstb.1996.0129

[b3] Posner M. & Snyder C. Attention and cognitive control. In Information Processing and Cognition: The Loyola Symposium, Solso, R. ed., Hillsdale, NJ: L. Erlbaum Assoc (1975).

[b4] Shallice T. & Burgess P. The domain of supervisory processes and temporal organization of behaviour. Philos. Trans. R. Soc. Lond B Biol. Sci. 351, 1405–1411 (1996). 8941952 10.1098/rstb.1996.0124

[b5] Botvinick M., Nystrom L. E., Fissell K., Carter C. S. & Cohen J. D. Conflict monitoring versus selection-for-action in anterior cingulate cortex. Nature 402(6758), 179–81 (1999). 10647008 10.1038/46035

[b6] Selemon L. D. & Goldman-Rakic P. S. Common cortical and subcortical targets of the dorsolateral prefrontal and posterior parietal cortices in the rhesus monkey: evidence for a distributed neural network subserving spatially guided behavior. J Neurosci 8, 4049–4068 (1988). 2846794 10.1523/JNEUROSCI.08-11-04049.1988 PMC6569486

[b7] Opris I. & Bruce C. J. Neural circuitry of judgment and decision mechanisms. Brain Res. Rev. 48, 509–526 (2005). 15914255 10.1016/j.brainresrev.2004.11.001

[b8] Fuster J. M. & Bressler S. L. Cognit activation: a mechanism enabling temporal integration in working memory. Trends in Cognitive Sciences 16(4), 207–218 (2012). 22440831 10.1016/j.tics.2012.03.005 PMC3457701

[b9] Alexander G. E., DeLong M. E. & Strick P. L. Parallel organization of functionally segregated circuits linking basal ganglia and cortex. Ann. Rev. Neurosci. 9, 357–381 (1986). 3085570 10.1146/annurev.ne.09.030186.002041

[b10] Opris I., Hampson R. E., Stanford T. R., Gerhardt G. A. & Deadwyler S. A. Neural activity in frontal cortical cell layers: evidence for columnar sensorimotor processing. J. Cogn. Neurosci. 23, 1507–1521 (2011). 20695762 10.1162/jocn.2010.21534 PMC3110724

[b11] Goodale M. A. & Milner A. D. Separate visual pathways for perception and action. Trends Neurosci. 15(1), 20–25 (1992). 1374953 10.1016/0166-2236(92)90344-8

[b12] Ungerleider L. G. & Mishkin M. Two cortical visual systems. In: Analysis of Visual Behavior (Ingle, D. J., Goodale, M. A.,Mansfield, R. J. W., eds), pp 549–586. Cambridge, MA, MIT (1982).

[b13] Rao S. C., Rainer G. & Miller E. K. Integration of what and where in the primate prefrontal cortex. Science 276, 821–824 (1997). 9115211 10.1126/science.276.5313.821

[b14] Middleton F. A. & Strick P. L. Basal-ganglia ‘projections’ to the prefrontal cortex of the primate. Cereb Cortex 12(9), 926–935 (2002). 12183392 10.1093/cercor/12.9.926

[b15] Hoover J. E. & Strick P. L. Multiple output channels in the basal ganglia. Science 259(5096), 819–821 (1993). 7679223 10.1126/science.7679223

[b16] McFarland N. R. & Haber S. N. Thalamic relay nuclei of the basal ganglia form both reciprocal and nonreciprocal cortical connections, linking multiple frontal cortical areas. J. Neurosci. 22(18), 8117–8132 (2002). 12223566 10.1523/JNEUROSCI.22-18-08117.2002 PMC6758100

[b17] Swadlow H. A., Gusev A. G. & Bezdudnaya T. Activation of a cortical column by a thalamocortical impulse. J Neurosci. 22, 7766–7773 (2002). 12196600 10.1523/JNEUROSCI.22-17-07766.2002 PMC6757983

[b18] Opris I. *et al.* Closing the loop in primate prefrontal cortex: Inter-laminar processing. Frontiers Neurosci. 6, 88 (2012). 10.3389/fncir.2012.00088 PMC3504312 23189041

[b19] Opris I., Hampson R. E., Gerhardt G. A., Berger T. W. & Deadwyler S. A. Columnar processing in primate pFC: Evidence for executive control microcircuits. J. Cogn. Neuro. 24(12), 2334–2347 (2012). 10.1162/jocn_a_00307 PMC3754813 23016850

[b20] Kritzer M. F. & Goldman-Rakic P. S. Intrinsic circuit organization of the major layers and sublayers of the dorsolateral prefrontal cortex in the rhesus monkey. J. Comp Neurol. 359, 131–143 (1995). 8557842 10.1002/cne.903590109

[b21] Rao S. G., Williams G. V. & Goldman-Rakic P. S. Isodirectional tuning of adjacent interneurons and pyramidal cells during working memory: evidence for microcolumnar organization in PFC. J. Neurophysiol. 81, 1903–1916 (1999). 10200225 10.1152/jn.1999.81.4.1903

[b22] Hubel D. H. & Wiesel T. N. Anatomical demonstration of columns in the monkey striate cortex. Nature 221, 747–750 (1969). 4974881 10.1038/221747a0

[b23] Takeuchi D., Hirabayashi T., Tamura K. & Miyashita Y. Reversal of interlaminar signal between sensory and memory processing in monkey temporal cortex. Science 331, 1443–1447 (2011). 21415353 10.1126/science.1199967

[b24] Weiler N., Wood L., Yu J., Solla S. A. & Shepherd G. M. Top-down laminar organization of the excitatory network in motor cortex. Nat. Neurosci. 11, 360–366 (2008). 18246064 10.1038/nn2049 PMC2748826

[b25] Mountcastle V. B. The columnar organization of the neocortex. Brain 120(4), 701–722 (1997). 9153131 10.1093/brain/120.4.701

[b26] Buxhoeveden D. P. & Casanova M. F. The minicolumn hypothesis in neuroscience. Brain 125, 935–951 (2002). 11960884 10.1093/brain/awf110

[b27] Jones E. G. Microcolumns in the cerebral cortex. Proc Natl Acad Sci USA 97, 5019–5021 (2000). 10805761 10.1073/pnas.97.10.5019 PMC33979

[b28] Rakic P. Confusing cortical columns. Proc Natl Acad Sci USA 105, 12099–12100 (2008). 18715998 10.1073/pnas.0807271105 PMC2527871

[b29] Casanova M. F., El-Baz A., Vanbogaert E., Narahari P. & Switala A. A topographic study of minicolumnar core width by lamina comparison between autistic subjects and controls: possible minicolumnar disruption due to an anatomical element in-common to multiple laminae. Brain Pathol. 20(2), 451–458 (2010). 19725830 10.1111/j.1750-3639.2009.00319.x PMC8094785

[b30] Di Rosa E., Crow T. J., Walker M. A., Black G. & Chance S. A. Reduced neuron density, enlarged minicolumn spacing and altered ageing effects in fusiform cortex in schizophrenia. Psychiatry Res. 166(2–3), 102–15 (2009). 19250686 10.1016/j.psychres.2008.04.007

[b31] Miller E. K. & Cohen J. D. An integrative theory of prefrontal cortex function. Annu. Rev. Neurosci. 24, 167–202 (2001). 11283309 10.1146/annurev.neuro.24.1.167

[b32] Santos L., Opris I., Fuqua J., Hampson R. E. & Deadwyler S. A. A novel tetrode microdrive for simultaneous multi-neuron recording from different regions of primate brain. J Neurosci Methods 205(2), 368–374 (2012). 22326226 10.1016/j.jneumeth.2012.01.006 PMC3342772

[b33] Wilson F. A., O'Scalaidhe S. P. & Goldman-Rakic P. S. Dissociation of object and spatial processing domains in primate prefrontal cortex. Science 260, 1955–1958 (1993). 8316836 10.1126/science.8316836

[b34] Barbas H., Hilgetag C. C., Saha S., Dermon C. R. & Suski J. L. Parallel organization of contralateral and ipsilateral prefrontal cortical projections in the rhesus monkey. BMC Neurosci 6, 32 (2005). 15869709 10.1186/1471-2202-6-32 PMC1134662

[b35] Hampson R. E. *et al.* Facilitation and Restoration of Cognitive Function in Primate Prefrontal Cortex by a Neuroprosthesis that Utilizes Minicolumn-Specific Neural Firing. J Neural Eng 9(5), 056012 (2012). 22976769 10.1088/1741-2560/9/5/056012 PMC3505670

[b36] Fuster J. M. Jackson and the frontal executive hierarchy. International Journal of Psychophysiology 64, 106–107 (2007). 16959349 10.1016/j.ijpsycho.2006.07.014

[b37] Opris I., Barborica A. & Ferrera V. P. Microstimulation of Dorsolateral Prefrontal Cortex Biases Saccade Target Selection. J Cogn Neurosci 17, 893–904 (2005). 15969908 10.1162/0898929054021120

[b38] Hopfield J. J. Neural networks and physical systems with emergent collective computational abilities. Proc Natl Acad Sci USA. 79(8), 2554–2558 (1982). 6953413 10.1073/pnas.79.8.2554 PMC346238

[b39] Hof B., de Lozar A., Kuik D. J. & Westerweel J. Repeller or Attractor? Selecting the Dynamical Model for the Onset of Turbulence in Pipe Flow. Physical Review Letters 101, 214501–4 (2008). 19113412 10.1103/PhysRevLett.101.214501

[b40] Buffalo E. A., Fries P., Landmanc R., Buschman T. J. & Desimone R. Laminar differences in gamma and alpha coherence in the ventral stream. Proc. Natl. Acad. Sci. U.S.A 108, 11262–67 (2011). 21690410 10.1073/pnas.1011284108 PMC3131344

[b41] Buschman T. J., Denovellis E. L., Diogo C., Bullock D. & Miller E. K. Synchronous oscillatory neural ensembles for rules in the prefrontal cortex. Neuron 76(4), 838–846 (2012). 23177967 10.1016/j.neuron.2012.09.029 PMC3907768

[b42] Berger T. W. *et al.* A cortical neural prosthesis for restoring and enhancing memory. J. Neural Eng. 8(4), 046017 (2011). 21677369 10.1088/1741-2560/8/4/046017 PMC3141091

[b43] Dobbs D. Schizophrenia: The making of a troubled mind. Nature 468, 154–156 (2010). 21068803 10.1038/468154a

[b44] Casanova M. F., Buxhoeveden D. & Gomez J. Disruption in the inhibitory architecture of the cell minicolumn: implications for autism. Neuroscientist 9(6), 496–507 (2003). 14678582 10.1177/1073858403253552

[b45] Brennan A. R. & Arnsten A. F. Neuronal mechanisms underlying attention deficit hyperactivity disorder: the influence of arousal on prefrontal cortical function. Ann. N. Y. Acad. Sci. 1129, 236–245 (2008). 18591484 10.1196/annals.1417.007 PMC2863119

[b46] Tomasi D. *et al.* Disrupted functional connectivity with dopaminergic midbrain in cocaine abusers. PLoS One 5(5), e10815 (2010). 20520835 10.1371/journal.pone.0010815 PMC2876035

[b47] Wang M. *et al.* Neuronal basis of age-related working memory decline. Nature 476, 210–213 (2011). 21796118 10.1038/nature10243 PMC3193794

[b48] Duncan J., Johnson R., Swales M. & Freer C. Frontal lobe deficits after head injury: Unity and diversity of function. Cognitive Neuropsychology 14, 713–741 (1997).

[b49] Shallice T. & Burgess P. W. Deficits in strategy application following frontal lobe damage in man. Brain 114(2), 727–741 (1991). 2043945 10.1093/brain/114.2.727

[b50] Hampson R. E., Coates T. D. Jr, Gerhardt G. A. & Deadwyler S. A. Ceramic-based micro-electrode neuronal recordings in the rat and monkey. Proceedings of the Annual International Conference of the IEEE Engineering in Medicine and BIology Society (EMBS) 25, 3700–3703 (2004).

