# Supplementary material for: Prefrontal cortical microcircuits bind perception to executive control
Source: Sci Rep. 2013 Jul 29;3:2285. doi: 10.1038/srep02285 (PMC3725477; doi:10.1038/srep02285)
Supplement: Supplementary Information — Supplementary Info [file srep02285-s1.pdf]

Prefrontal cortical microcircuits bind perception to executive control

Ioan Opris[1], Lucas Santos[1], Greg A. Gerhardt[2], Dong Song[3] Theodore W. Berger[3] Robert E. Hampson[1] and Sam A. Deadwyler[1]

SUPPLEMENTARY FIGURES

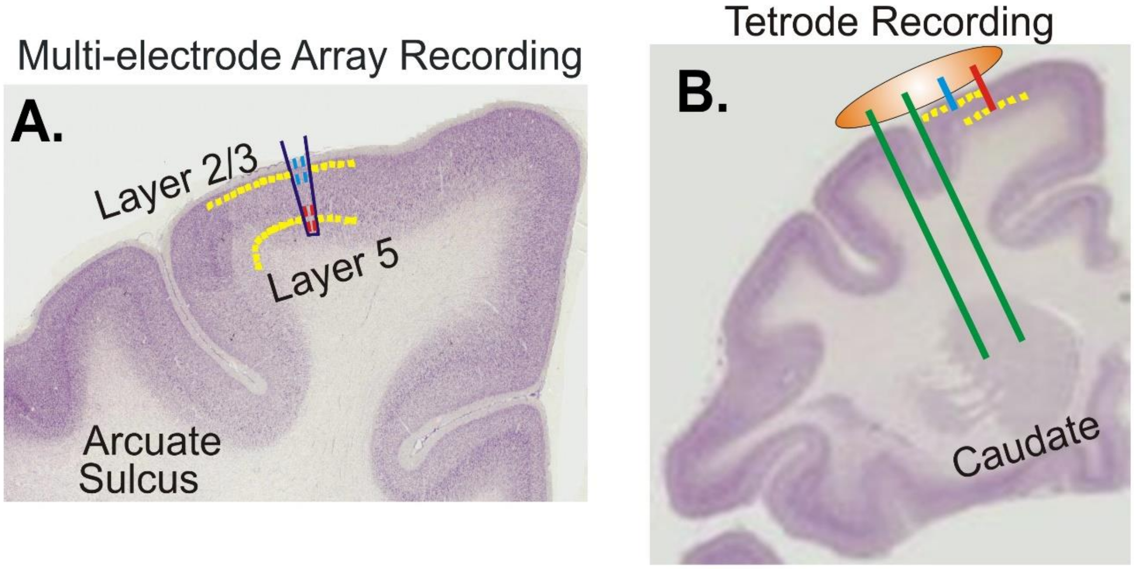

Supplementary Figure 1. Examples of conformal recording used for inter-laminar & cortico-striatal simultaneous recording. A. Coronal section in the prefrontal cortex showing the placement of W3 recording array (with inter-layer separation of 1,350 um) for inter-laminar-columnar recording. **B.** Similar display to show tetrode simultaneous recording in prefrontal cortical layers 2/3, 5, and caudate.

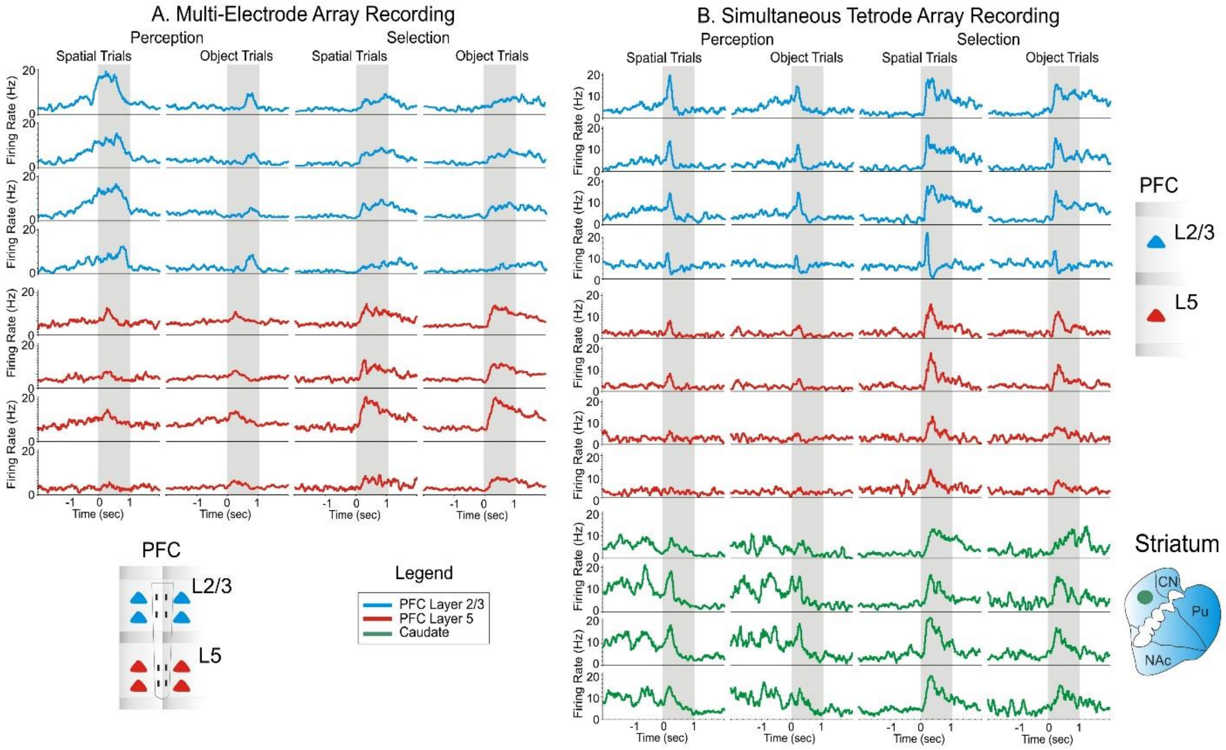

Supplementary Figure 2. Neural activity recorded with MEA array and with tetrode microdrive. A. Individual neuron peri-event histograms depicting the activity of prefrontal cells from layers 2/3 & 5 during perception (sample phase in DMS task) and executive selection (match phase). **B.** Comparison of neural firing in individual neuron peri-event histograms in prefrontal cells from layers 2/3 & 5 and caudate recorded simultaneously with the tetrode microdrive during perception (Sample phase in DMS task) and executive selection (Match phase).

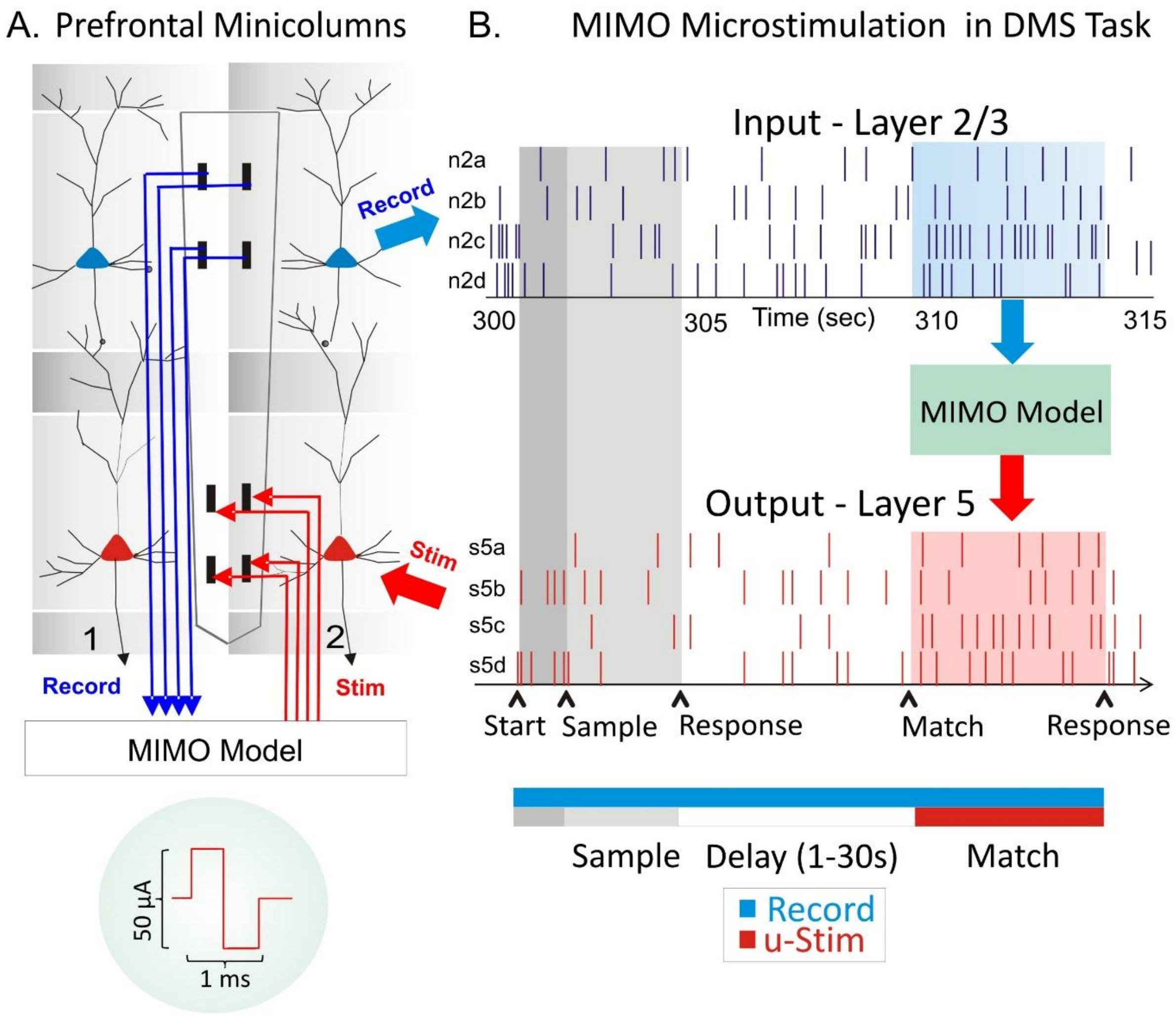

Supplementary Figure 3. Inter-laminar MIMO microstimulation during DMS task A. Diagram illustrating the interfacing of MIMO model with conformal MEAs shown in Fig 5 between L2/3 and L5 during task performance. Electrical stimulation was delivered to MEA pads in L5 via patterns of pulses (biphasic) recorded from the same L5 locations on successful trials by the MIMO model. **B**. Firing of L2/3 and L5 located columnar neurons was recorded online and fed to MIMO model shown in **A**. Shaded areas indicate the time epoch from Match Presentation to Match Response execution during DMS trial, and the stimulation pattern in L5 together with the recorded firing pattern in L2/3. Reproduced with permission from Ref (18).

SUPPLEMENTARY METHODS:

All animal procedures were reviewed and approved by the Institutional Animal Care and Use Committee of Wake Forest University, in accordance with U.S. Department of Agriculture, International Association for the Assessment and Accreditation of Laboratory Animal Care, and National Institutes of Health guidelines.

Behavioral Response: The rhesus macaques utilized as subjects (n=4) were trained for at least 2 years to perform a well characterized, custom-designed visual DMS task (Fig. 1C)[18,51]. Animals were seated in primate chairs in front of a display screen (Fig 1A) during performance of the task. Right limb (arm) position was tracked via a UV-fluorescent reflector affixed to the back of the wrist which was illuminated with a 15 W UV lamp tracked by a small LCD camera positioned 30 cm above the hand. Horizontal position of the illuminated target was computed from the video image using a Plexon Cineplex scanner connected to a behavioral control computer. Hand position and movement was digitized and displayed as a bright yellow cursor on the projection screen.

Rule-based Delayed-Match-to-Sample (DMS) Task: Trials were initiated by the animal placing the cursor inside "Start rule" signal which consisted of a yellow 3” diameter circle for object trials or a 3” square for spatial trials) which was randomly illuminated in one of the 9 geometrically related spatial positions on the screen (Fig 1C). Placement of the cursor into the Start ring produced a randomly chosen “Sample” image from a continually altered database of 10,000 trial-unique “clip-art” images, displayed at one of 9 screen positions for a maximum of 2s (“Sample Phase”). The animal was required to place the cursor on the

Sample image (Sample Response) for 100-300 ms to initiate the subsequent Delay phase in which the screen went blank for a 1-40 sec delay interval, the duration of which was randomly selected on each trial. Upon timeout of the Delay interval, the Match phase of the task was initiated in which there was immediate screen display of 2–4 images at different, randomly selected spatial locations, one of which was the Sample image, and 1-3 were Non-match "distracter" images presented randomly at the other 8 screen locations. A response of placing the cursor into the Sample image constituted a correct "Match Response" which was rewarded immediately by a drop of juice delivered via a sipper tube located near the animal's mouth with simultaneous blanking of the screen. Placement of the cursor into a non-Sample image was registered as a 'Non-match error' and caused the screen to blank without delivery of juice reward. All trials were separated by a 10.0 sec inter-trial interval (ITI) before re-presentation of the Start Signal. Neither of the Sample or non-Match (distracter) images was presented on more than a single trial within the entire session (150–200 trials), and the 5000 item image selection buffer was replaced with new images every month[52]. All subjects were trained to overall performance levels of 75% correct on the above DMS task parameters. Daily performance as a function of number of images in the Match phase and Delay epoch is shown in Fig. 1D.

Surgery: Animals were surgically prepared with cylinders for attachment of a microelectrode manipulator over the specified brain regions of interest. During surgery animals were anesthetized with ketamine (10 mg/kg), then intubated and maintained with isoflurane (1-2 % in oxygen 6 l/min). The recording cylinders (Crist Instruments, Hagerstown, MD) were placed over 11 mm diameter craniotomies for electrode access.11,

32 Craniotomy sites were selected to overlie the stereotaxic coordinates of the Frontal Cortex (25 mm anterior relative to Interaural line and 12 mm lateral to midline/vertex) in the caudal region of the Principal Sulcus, the dorsal limb of Arcuate Sulcus in area 8 and the dorsal part of premotor area 6 (Fig. 1a), areas previously shown by PET imaging to become activated during task performance[51]. Two titanium posts were secured to the skull for head restraint with titanium steel screws embedded in bone cement. Following surgery, animals were given 0.025 mg/kg buprenorphine for analgesia and penicillin to prevent infection. Cylinders were disinfected thrice weekly with Betadine during recovery and daily during recording.

Electrophysiological Recording and Microstimulation. Electrophysiological procedures and analysis software for 64 channel simultaneous recordings utilized the MAP Spike Sorter by Plexon, Inc (Dallas, TX). Two types of recording were performed: a) one using the tetrode recording simultaneously in PFC layers and caudate-putamen[32] and b) one using customized ceramic electrodes, designed and manufactured collaboratively with Dr. Greg Gerhardt (Center for Microelectrode Technology – CenMet, Lexington, KY) at the University of Kentucky,[50,53] which consisted of etched printed circuit platinum recording pads, employed for recording of single neuron activity in PFC inter-laminar microcircuits[10,50]. The model W3 configured Kentucky probe was specially designed such that recording pads 1-4 recorded activity from neurons in the infra-granular layer 5 while pads 5-8 separated vertically by 1350um simultaneously recorded neuron activity in the supra-granular layer 2/3[10,18,35,50]. Extracellular action potentials were isolated and analyzed with respect to firing during specific events within DMS trials (Figs 1-4). Recordings from

multiple pads in designated locations on the MEAs were analyzed by a nonlinear model previously perfected for assessing and extracting spatiotemporal multineuron firing patterns in PFC using the same MEAs and to deliver task-contingent electrical stimulation[35] to layer 5 in the same pattern as recorded during correct trial performance. Stimulation (see Supplementary Figure S3)[18,35] consisted of 1.0 ms bipolar pulses (50 uA) delivered to layer 5 recording locations following presentation of the Match phase screen at the time predicted by recording from Layer 2/3 cells during the completion of the movement (Figure 4).

Data Analysis: Behavioral assessments of task performance (% correct trials) were computed for each animal (n=4) and related to individual trials in which simultaneous electrophysiological recordings of conformal MEA recorded multiple single neuron activity was compared for Sample (perception) and Match (selection) phases of the task.[18] A MAP Spike Sorter (Plexon, Inc) was used to discriminate and analyze single-neuron waveforms with the multineuron acquisition processor and NeuroExplorer software (NEX Technologies, Littleton, MA). Cell types were identified by the presence (or absence) of significant ($z > 3.09$, $P < 0.001$) firing rate changes (see Statistical Analyses, below) in rastergrams of neural firing (dots) on single trials (rows) and in peri-event histograms derived for intervals of ± 2.0 s around specific task events (Figs 2,3).

Neural firing was classified according to recording pads placed in particular cortical layers on the conformal MEAs[18]. To account for neuronal responses in terms of columnar microcircuit organization frontal cortical neurons were characterized by 1) layer specific firing, and 2) whether the firing pattern was modulated by presentation of the Sample/Match phase of the DMS task[18,35]. Analyses were performed for triplets of neurons recorded

simultaneously in different cortical layers (L2/3 and L5) and in caudate based on the distances corresponding to the vertical and horizontal separations between sets of recording pads on the ceramic MEA electrodes (Fig. 1S)[10,50]. Cross-correlation (CCH) was calculated for pairs of cells between prefrontal layer 5 and caudate nucleus[16-18].

Statistical Analyses. Standard scores, z = [peak - baseline firing rate]/SD baseline firing rate, were calculated for individual cell firing during each DMS task event. Firing rate was analyzed in 25 ms bins for ± 2.0 s surrounding task events. Neurons were only included in the analysis if their firing rates were significantly elevated from Pre-Match presentation baseline (Z scores, ANOVA F test $p < 0.01$, Figs 2&3). Statistical Analyses were also used to test whether there were inter-laminar differences in cell firing within different layers (i.e. layer 2/3 vs. layer 5) for specific events including mostly Match phase activation patterns.

Tuning Plots: For each analyzed neuron triplet (L2/3, L5 and caudate), firing on the same trials was aligned to presentation of the match target position selected. Directionality was assigned according to 8 different "clock" directions corresponding to the location of the match image around the periphery of the screen, yielding 0°, 45°, 90°, 135°, 180°, 225°, 270°, 315° & 360° movement directions (from center of screen). Mean firing rate after match presentation but immediately prior to match response (i.e. 0.0-1.0 s) for each response position was calculated and represented in polar coordinates as a tuning plot[21,55]. The directional bias for a given cell was revealed by the response locations with the highest mean firing rate *and* by the direction of tuning vectors computed during the match epoch (Fig 2, polar plots). To assess the spatial preference we compared statistically both the amplitude (Fig 3E&F) and the direction (Fig. 4A-C) of the average tuning vectors. Similarly, tuning vectors were plotted for percent correct performance under stimulation vs. no

stimulation conditions. To quantify similarity of tuning polygonal contours we used overlap values between 0 (totally dissimilar) and 1 (identical, no statistical significance) and Rayleigh test for statistical significance[55].

SUPPLEMENTARY REFERENCES:

51. Deadwyler, S.A., Porrino, L.P., Siegel, J.E. & Hampson, R.E. Systemic and nasal delivery of orexin-A (hypocretin-1) reduces the effects of sleep deprivation on cognitive performance in nonhuman primates. J Neurosci 27, 14239–14247 (2007).

52. Hampson, R.E., Pons, T.P., Stanford, T.R. & Deadwyler, S.A. Categorization in the monkey hippocampus: a possible mechanism for encoding information into memory. Proc Natl Acad Sci USA 101, 3184–3189 (2004).

53. Moxon, K. A., Leiser, S. C., Gerhardt, G. A., Barbee, K. A., & Chapin, J. K. Ceramic based multisite electrode arrays for chronic single-neuron recording. IEEE Transactions on Biomedical Engineering, 51, 647–656 (2004).

54. Hampson, R.E., Opris, I. & Deadwyler, S.A. Neural correlates of fast pupil dilation in nonhuman primates: Relation to behavioral performance and cognitive workload. Behav Brain Res 212, 1–11 (2010).

55. Opris, I., Barborica, A. & Ferrera, V.P. Effects of Electrical Microstimulation in Monkey Frontal Eye Field on Saccades to Remembered Targets. Vision Research 45, 3414-3429 (2005).
